# Infertility perceptions and associated factors among Korean undergraduate students: a cross-sectional study

**DOI:** 10.3389/fpubh.2026.1741644

**Published:** 2026-07-08

**Authors:** Miok Kim, Hyeon Sik Chu, Su Jeong Yi

**Affiliations:** College of Nursing, Dankook University, Cheonan, Republic of Korea

**Keywords:** family values, gender roles, infertility, perception, university students

## Abstract

**Introduction:**

South Korea faces a declining total fertility rate (0.72 in 2023) alongside rising infertility rates. Infertility is often interpreted within a pronatalist sociocultural context emphasizing marriage, parenthood, and traditional gender role expectations. Despite increasing infertility rates, little research has focused on how young adults—particularly university students—perceive infertility.

**Methods:**

A descriptive correlational study was conducted with 159 unmarried university students (mean age = 21.6 years; 79.2% women). Participants completed an online survey measuring infertility perception (using the Infertility Perception Scale for Women, IPS-W), traditional gender stereotypes, and family values. Data were analyzed using independent t-tests, one-way ANOVA, Pearson correlation, and multiple regression.

**Results:**

The mean infertility perception score was 37.97 (SD = 8.23), indicating a moderately positive perception. Male students, those with religious affiliation, and those without exposure to media on assisted reproductive technology held significantly more negative perceptions (*p* < 0.05). Infertility perception was positively correlated with traditional gender stereotypes (*r* = 0.541) and family-oriented values emphasizing marriage and childbearing (*r* = 0.584). Multiple regression revealed that gender stereotypes (*β* = 0.370, (*p* < 0.001) and family values (*β* = 0.457, *p* < 0.001) together explained 49% of the variance in infertility perception (adjusted R^2^ = 0.49).

**Discussion:**

Stronger endorsement of traditional gender roles and family-oriented values that prioritize childbearing was associated with more negative perceptions of infertility. Integrating gender-sensitive and media-supported infertility education into university curricula and public health strategies may improve perception, reduce stigma, and foster healthier reproductive decision-making.

## 1 Introduction

South Korea has experienced a dramatic decline in its total fertility rate (TFR), decreasing from 1.24 in 2015 to 0.84 in 2020, and reaching 0.72 in 2023 and 0.75 in 2024 (1). In this demographic context, infertility is no longer merely a personal issue but has emerged as a significant social concern. National data indicate that the number of individuals diagnosed with infertility increased from 227,922 in 2018 to 238,601 in 2022, representing a 4.7% rise over 5 years. During the same period, the number of patients undergoing infertility treatments increased by 16.0%. Notably, total annual medical expenditures for infertility care nearly doubled, rising from 124.5 billion KRW in 2018 to 244.7 billion KRW in 2022. This increase reflects not only greater service utilization but also a substantial rise in per capita medical costs, which increased by 87.7% over the same period (2). These figures underscore the growing socioeconomic burden of infertility. This highlights the need to foster positive perceptions and reduce stigma, ensuring that affected individuals can seek timely treatment without psychological barriers.

As infertility becomes more visible in public discourse, societal perceptions and attitudes toward infertility play an increasingly important role in shaping the experiences of affected individuals. Negative or stigmatizing perceptions may exacerbate psychological distress and social isolation among individuals with infertility (3, 4). Moreover, perceptions of infertility vary according to sociocultural factors such as gender, socioeconomic status, religion, and race, and even within the same society, these perceptions can differ based on one’s cultural and economic background (5, 6). Therefore, it is necessary to assess perception of infertility across diverse population groups and examine the variations that exist among them.

Notably, perceptions and attitudes toward infertility are closely linked to individuals’ value systems, such as gender stereotypes and family values (7). Gender stereotypes, defined as cognitive generalizations that assign specific roles and characteristics based on gender (8), can restrict one’s thoughts and behaviors in various situations (9). In Korean society, where Confucian traditions are deeply rooted, such stereotypes tend to reinforce rigid gender biases (10), particularly by emphasizing women’s primary responsibility for childbearing and caregiving as central components of womanhood. Family values, referring to beliefs and attitudes about the family (11), directly influence perceptions related to children, childbirth, and parenting (12). When individuals hold strong traditional gender stereotypes and family values, there is a tendency to perceive infertility as a personal failure, particularly of women (13), which may lead to more negative perceptions and maladaptive coping responses regarding infertility. Despite this, there remains a lack of empirical research examining how gender stereotypes and family values influence infertility perceptions.

In the Korean cultural context, family values often emphasize the importance of childbearing, and traditional gender norms may view motherhood as a central aspect of womanhood. In East Asian societies, including South Korea and Japan, strong pronatalist values and family-centered ideologies emphasize maintaining family lineage and fulfilling social and ethical responsibilities through having descendants (14). In such contexts, infertility may be perceived not merely as an individual health issue but as a concern affecting the entire family and as a failure to meet societal expectations (4, 15). Unlike Western countries, where reproductive health is more often framed as an individual right within broader discussions of reproductive autonomy, gender equality, and social welfare (16), reproductive issues in South Korea have largely been framed within the context of national demographic crisis and population policy since the early 2000s. In response, the government enacted the Framework Act on Low Birth Rate and Aging Society in 2005 and has implemented successive five-year national plans to address low fertility (17). These policies have primarily focused on promoting childbirth and expanding state support for pregnancy, childcare, and work–family balance. Although recent plans have broadened their scope to include employment, housing, and life-course welfare measures, policy discourse has largely centered on reversing fertility decline rather than prioritizing reproductive health and individual rights (18). This policy orientation reinforces a pronatalist view, positioning infertility as part of a national demographic strategy rather than a personal health issue, which may further shape societal perceptions of infertility. Therefore, understanding perceptions of infertility requires consideration of the unique cultural context of Korean society, which places a high value on childbirth and family continuity, often viewing childbearing as a central responsibility of married women.

University students are in a critical developmental stage where they form their identity, life perspectives, and personal values (9). As young adults at a formative stage of life, their health perceptions and attitudes, including those toward infertility, can provide early insight into emerging societal trends and inform interventions to promote healthy reproductive behaviors. However, most existing studies on infertility perceptions have focused on married individuals, those with infertility experiences, or middle-aged adults (4, 19), leaving research on university students limited.

This study draws on a sociocultural and social constructionist framework (13, 20–22). From this perspective, perceptions of infertility are shaped by cultural norms, gender role expectations, and family-centered values that are deeply embedded in Korean society, and the meaning attached to infertility is not a fixed biological fact but something actively built through cultural norms and institutional discourse, so how infertility is perceived depends heavily on its social context. Ecological systems theory (20) (pp. 21–26) describes individual development as nested within several layers of context, from the immediate family environment (the microsystem) to broader societal structures and policies (the macrosystem), and we use this framework here to situate infertility perception within family norms, gender role structures, and national pronatalist policy in Korea. Gender system theory (21) adds that institutionalized gender arrangements shape individual beliefs, which led us to expect that stronger adherence to traditional gender roles would be associated with more negative perceptions of infertility. Based on these perspectives, we expected that individuals internalize pronatalist values and gendered expectations in ways that shape whether infertility is understood as a medical condition or as a personal failure.

Despite the increasing prevalence and societal salience of infertility in Korean society, little is known about how young adults perceive it within such a sociocultural context. Therefore, this study aims to examine the levels of infertility perception, gender stereotypes, and family values among university students, analyze the relationships among these variables, and identify the factors influencing infertility perception. The findings are intended to serve as a basis for developing educational programs and policies tailored to improve infertility perception among young adults.

## 2 Methods

### 2.1 Study design

This descriptive correlational study aimed to identify the levels of infertility perception, gender stereotypes, and family values among college students and to examine the factors influencing infertility perception.

### 2.2 Study participants

The participants were unmarried college students aged 19 to 29 years who had access to the internet and were able to complete an online survey. Participants were eligible if they understood the purpose of the study and provided informed consent. The study was restricted to unmarried individuals for two reasons: first, most prior research on infertility perception has focused on married couples or individuals with personal infertility experience (4, 19), leaving unmarried young adults understudied; second, excluding married individuals allowed us to control for the potential confounding influence of direct personal infertility experience or marital pressure on perception scores. Individuals were excluded if they were married, cohabiting with a partner, or currently taking medication for chronic illnesses or mental health conditions. Although a small proportion of participants (3.8%) reported currently receiving medical treatment, they were retained in the study because their conditions were minor and did not require psychiatric medications or treatments for severe chronic illnesses that could potentially affect the study outcomes.

The required sample size for statistical analysis was calculated using G*Power version 3.1.9.4. With a significance level of 0.05, statistical power of 0.80, and an effect size of 0.15 (Cohen’s f^2^, medium), and considering 14 predictor variables (sociodemographic variables, two primary study variables, and the dependent variable) for multiple linear regression analysis, the minimum required sample size was 139. To account for a potential 10% dropout rate, the target sample size was set at 159 participants.

### 2.3 Data collection

Data were collected from January 20 to March 10, 2025, using an online survey platform. Although the data collection period spanned approximately 50 days, no major infertility-related policy changes, public health events, or significant media incidents occurred during this period that could have systematically altered participants’ perceptions of infertility. The survey assessed stable attitudinal constructs (infertility perception, gender stereotypes, and family values) rather than responses to specific current events, further supporting the assumption of a relatively consistent social environment across participants. The survey link was shared via online community boards frequented by college students. On the first page of the survey, participants were provided with detailed information about the study and were required to indicate their consent before proceeding. After providing electronic informed consent, they completed the survey anonymously.

### 2.4 Measures

#### 2.4.1 Sociodemographic characteristic of participants

The participants’ sociodemographic characteristics, presented in Table 1, included age, gender, grade (academic year), religion, number of siblings (including oneself), field of study, experience of sex education after entering university, experience of infertility and reproductive health education, taking courses related to values on marriage and childbearing (since university enrollment), having acquaintances who have received infertility treatment, and exposure to media on assisted reproductive technology (ever experienced, with no specific time frame restricted). In addition, marital and relationship status and current receipt of medical treatment were assessed as eligibility-related items and are reported descriptively in the Results section.

**Table 1:** Infertility perception by sociodemographic characteristics (*N* = 159).

Variables	Category	n (%) or M ± SD	Infertility perception	
			M ± SD	t (*p*)
Age (yr)		21.57 ± 2.03		
Gender	Male	33(20.8)	42.69 ± 8.46	3.861 (<0.001)
	Female	126(79.2)	36.73 ± 7.73	
Grade	1	30(18.9)	37.36 ± 7.59	1.159 (0.329)
	2	38(23.9)	38.21 ± 8.05	
	3	53(33.3)	39.39 ± 7.94	
	4	38(23.9)	36.23 ± 9.18	
Religion	Having religion	39(20.8)	40.61 ± 7.54	2.338 (0.021)
	No religion	126(79.2)	37.11 ± 8.29	
Number of Siblings (Including Oneself)	1	34(21.4)	39.29 ± 8.86	0.944 (0.391)
	2	94(59.1)	37.98 ± 8.14	
	3 or more	31(19.5)	36.48 ± 7.77	
Field of study	Humanities and Social Sciences	13(8.2)	36.61 ± 7.53	0.894 (0.469)
	Engineering	15(9.4)	36.80 ± 9.74	
	Natural Sciences	63(39.6)	38.69 ± 8.54	
	Health and Medical Sciences	62(39.0)	38.30 ± 7.70	
	Arts and Physical Education	6(3.8)	32.83 ± 7.83	
Experience of sex education after entering university	Yes	57(35.8)	38.03 ± 8.24	0.069 (0.945)
	No	102(64.2)	37.94 ± 8.26	
Experience of infertility and reproductive health education	Yes	54(34.0)	36.72 ± 6.81	−1.380 (0.170)
	No	105(66.0)	38.61 ± 8.83	
Taking courses related to values on marriage and childbearing	Yes	35(22.0)	38.02 ± 7.37	0.044 (0.965)
	No	124(78.0)	37.95 ± 8.48	
Having acquaintances who have received infertility treatment	Yes	20(12.6)	38.25 ± 9.92	0.159 (0.874)
	No	139(87.4)	37.93 ± 8.00	
Exposure to Media on Assisted Reproduction	Yes	122(76.7)	37.17 ± 7.65	−2.261 (0.025)
	No	37(23.3)	40.62 ± 9.53	
Currently Receiving Medical Treatment	Yes	6(3.8)	35.83 ± 7.02	−0.648 (0.518)
	No	153(96.2)	38.05 ± 8.28	

#### 2.4.2 Infertility perception

In this study, infertility perception was measured using the Infertility Perception Scale for Women (IPS-W) developed by Kim and Ban (4). This instrument consists of 21 items across four subdomains and is rated on a 4-point Likert scale ranging from 1 (“Strongly disagree”) to 4 (“Strongly agree”). The four subdomains are: (1) perceived feelings (6 items), capturing emotional responses such as loss, anxiety, and despair associated with infertility; (2) personal stigma (8 items), reflecting the extent to which individuals view infertility as a personal failure or a problem difficult to disclose; (3) social stigma (3 items), representing beliefs that infertility carries societal stigma and leads to relational withdrawal; and (4) acceptance (4 items), reflecting the degree to which infertility is perceived as a surmountable condition that can be managed as a couple. Sample items include statements such as, ‘*I feel a sense of loss if I cannot have a child,’* and *‘Infertility is a solvable issue.’* A negative perception reflects viewing infertility as a personal failure or a source of stigma, whereas a positive perception involves recognizing infertility as a medical condition that can be addressed through treatment and support. Participants were instructed to respond to the items from a hypothetical perspective (i.e., imagining themselves experiencing infertility). The total score ranges from 21 to 84, with higher scores indicating more negative perceptions of infertility. The Cronbach’s alpha was 0.90 at the time of the original development (4), with subdomain-level Cronbach’s alpha values of 0.91 (perceived feelings), 0.85 (personal stigma), 0.77 (social stigma), and 0.60 (acceptance) (4). In the present study, the Cronbach’s alpha was 0.85 for the total sample and 0.83 specifically for the male participants (n = 33), indicating acceptable internal consistency across genders. The subdomain-level Cronbach’s alphas in this study were 0.89 for perceived feelings, 0.83 for personal stigma, 0.71 for social stigma, and 0.58 for acceptance. Although the reliability for the acceptance subdomain was relatively lower, it aligns closely with the baseline of 0.60 reported in the development study, which is considered acceptable given the small number of items (4 items) and the non-clinical status of the sample.

#### 2.4.3 Gender stereotypes

Gender stereotypes were assessed using a 33-item scale developed by Kim (10). The instrument comprises five subscales: familial gender roles, social gender roles, occupational and appearance-related gender roles, psychosocial gender roles, and intellectual gender roles. Sample items include statements such as, *‘It is better for women to take care of the home and family, while men work outside’*, and *‘Even if a woman has a job, she should take primary responsibility for household chores.’* Each item was rated on a 5-point Likert scale, with higher total scores indicating stronger gender stereotypes. In the original study (10), the Cronbach’s alpha values ranged from 0.70 to 0.83, and the reliability coefficient in the present study was 0.94.

#### 2.4.4 Family values

Family values were assessed using an instrument reconstructed and supplemented by Seo and Kim (23) based on previous studies. The tool consists of seven items, each rated on a 7-point Likert scale ranging from “strongly disagree” (1 point) to “strongly agree” (7 points). Sample items include questions such as, *‘Do you think having children is absolutely necessary in life?’* and *‘Do you consider becoming a parent to be a valuable life experience?’* Higher total scores indicate stronger traditional familism values, reflecting greater importance placed on having children and becoming parents (12). At the time of the tool’s development (23), Cronbach’s alpha values ranged from 0.77 to 0.84, and the reliability coefficient in the present study was 0.91.

### 2.5 Data analysis

The collected data were analyzed using SPSS version 26.0, with a significance level set at 0.05 and two-tailed tests. Descriptive statistics, including frequencies, percentages, means, and standard deviations, were used to present the participants’ sociodemographic characteristics and the levels of major variables. Differences in infertility perceptions according to sociodemographic characteristics were analyzed using independent t-tests and one-way ANOVA, with Scheffe’s method employed for *post hoc* comparisons. The relationships among family values, gender stereotypes, and infertility perceptions were examined using correlation coefficients. Factors influencing infertility perceptions were analyzed using multiple linear regression analysis.

### 2.6 Ethical considerations

This study was approved by the Institutional Review Board (IRB) of the author’s affiliated institution (Approval No. 2024–11–035-002), and all procedures were conducted in accordance with ethical standards for research involving human participants. Informed consent was obtained from all participants prior to data collection. Participants were fully informed about the study’s purpose, procedures, and any potential risks and benefits. They were also assured of their right to withdraw from the study at any time without penalty. All data collected were anonymized, and participants’ personal information was kept strictly confidential. As a token of appreciation, participants received a small financial incentive upon completion of the survey.

## 3 Results

### 3.1 Participants’ sociodemographic characteristics and associated differences in infertility perception

The mean age of participants was 21.57 years (SD = 2.03), and the majority were female (79.2%). Participants were evenly distributed across the four academic years, from freshman to senior. Most participants (79.2%) reported having no religious affiliation. Regarding academic majors, 39.6% were enrolled in natural sciences and 39.0% in health and medical sciences. The most commonly reported number of siblings (including the participant) was two, as reported by 59.1% of the respondents. A small proportion (3.8%) indicated that they were receiving treatment for a medical condition.

In terms of educational experiences, 35.8% had received sex education since entering university, 34.0% had received education related to infertility and reproductive health, and 22.0% had taken courses related to values regarding marriage and childbearing. Additionally, 12.6% reported having acquaintances who had undergone infertility treatment, and 76.7% had been exposed to media content related to assisted reproductive technologies (ART).

At the item level (not presented in tabular form), a large majority of participants agreed with the statements *‘Infertility is a solvable issue’* (81.2%), *‘If the couple shares the same perspective, infertility does not significantly affect married life’* (87.4%), and *‘Infertility should be accepted as a natural part of life’* (78.6%).”

The participants’ infertility perception scores ranged from 21 to 84, with a mean of 37.97 (SD = 8.23). Scores for gender stereotypes ranged from 33 to 165, with a mean of 69.85 (SD = 16.30). Family values scores ranged from 7 to 49, with a mean of 30.21 (SD = 10.78; Table 2).

**Table 2:** Descriptive statistics and correlations between infertility perception, gender role stereotypes, family values and their subdomains (*N* = 159).

Variables	M ± SD	1	2	3	4	5	6	7	8	9
1. Infertility Perception	37.97 ± 8.23	1								
2. Gender Stereotypes (Total)	69.85 ± 16.31	0.541**	1							
3. Social Gender Roles	7.23 ± 2.76	0.463**	0.728**	1						
4. Familial Gender Roles	12.68 ± 4.61	0.584**	0.739**	0.772**	1					
5. Occupational Gender Roles	16.26 ± 5.81	0.499**	0.890**	0.660**	0.600**	1				
6. Psychosocial Gender Roles	12.64 ± 5.32	0.408**	0.846**	0.558**	0.511**	0.688**	1			
7. Intellectual Gender Roles	8.24 ± 3.72	0.409**	0.776**	0.635**	0.522**	0.655**	0.670**	1		
8. Family Values (Total)	30.21 ± 10.78	0.584**	0.281**	0.288**	0.435**	0.223**	0.095	0.200*	1	
9. Value of Children	11.11 ± 5.32	0.546**	0.273**	0.263**	0.421**	0.189*	0.084	0.220**	0.925**	1
10. Value of Parenthood	19.11 ± 6.20	0.547**	0.255**	0.276**	0.396**	0.227**	0.093	0.159*	0.945**	0.751**

*p < 0.05, **p < 0.01.

Differences in infertility perception based on participants’ general characteristics were observed for gender, religious affiliation, and exposure to media related to ART. Specifically, male participants, those with a religious affiliation, and those who had not been exposed to such media tended to have more negative perceptions of infertility (Table 1).

### 3.2 Correlation between infertility perception, gender stereotypes and family values

Correlation analysis revealed significant associations between infertility perception, gender stereotypes, family values, and their respective subdomains (Table 2). Overall, stronger family values (*r* = 0.584, *p* < 0.001) and higher levels of traditional gender stereotypes (*r* = 0.541, *p* < 0.001) were significantly correlated with more negative infertility perceptions.

When examining the subdomains, both the value of children (*r* = 0.546, *p* < 0.001) and the value of parenthood (*r* = 0.547, *p* < 0.001) within family values showed strong positive correlations with negative infertility perceptions. Among the gender stereotype subdomains, familial gender roles exhibited the strongest correlation with infertility perception (*r* = 0.584, *p* < 0.001). Other subdomains, including occupational (*r* = 0.499, *p* < 0.001), social (*r* = 0.463, *p* < 0.001), intellectual (*r* = 0.409, *p* < 0.001), and psychosocial gender roles (*r* = 0.408, *p* < 0.001), were also significantly correlated with infertility perception, though to a slightly lesser extent.

### 3.3 Factors influencing infertility perception

This study conducted a multiple regression analysis to identify factors influencing perceptions of infertility. The analysis included key variables used in the study, as well as general characteristics that showed significant differences in the level of infertility perception - namely gender, religious affiliation, and exposure to media related to assisted reproductive technology. The results revealed that gender stereotypes (B = 0.187, *p* < 0.001) and family values (B = 0.349, *p* < 0.001) had statistically significant effects on infertility perception. This indicates that higher levels of gender stereotypes and stronger family values are associated with more negative perceptions of infertility. The explanatory power of the regression model was 49%, suggesting that these variables explain a considerable portion of the variance in infertility perception (Table 3).

**Table 3:** Factors influencing infertility perception (*N* = 159).

Variable	B	β	SE	t (*p*)
Constant	14.607		2.593	5.632(<0.001)
Gender stereotypes	0.187	0.370	0.032	5.867(<0.001)
Family values	0.349	0.457	0.046	7.652(<0.001)
Gender (Ref: Female)	1.056	0.052	1.248	0.846(0.399)
Religion (Ref: No Religion)	1.559	0.082	1.095	1.423(0.157)
Exposure to media on assisted reproduction	−1.117	−0.057	1.150	−0.971(0.333)

R^2^ = 0.51, Adjusted R^2^ = 0.49, *F* = 31.651, *p* < 0.001, Durbin-Watson = 1.885.

## 4 Discussion

In this study, the mean infertility perception score among university students was 37.97 (SD = 8.23), which was lower than the average score of 42.4 (SD = 9.1) reported among women diagnosed with infertility at the time of the tool’s development (4). Although these two groups differ in age, marital status, and direct experience with infertility, any of which could independently affect perception scores, this comparison is best treated as a rough point of reference rather than a direct equivalence, since no normative IPS-W data currently exist for unmarried young adults responding from a hypothetical perspective. With that caveat in mind, the lower score observed here suggests that university students may hold somewhat more positive perceptions of infertility than women directly facing it. This may partly reflect developmental stage: most university students have not yet experienced marriage or childbirth, so infertility may feel more like a distant societal issue than an immediate personal one.

As shown in the item-level results, the high agreement with statements characterizing infertility as a solvable issue or a natural part of life reflects a more optimistic view of infertility among this sample, which could be influenced by broader societal changes, including increased availability of treatment options.

The level of infertility perception among university students in this study significantly differed by gender, religious affiliation, and prior exposure to media content addressing issues related to assisted reproductive technology. Male students demonstrated more negative perceptions of infertility. This finding aligns with previous studies involving Korean university students, which reported that male students were more likely than females to express a strong desire to have children and to view parenthood as a central life goal (24, 25). In other words, men who place higher value on childbearing may perceive infertility as a greater threat, which could lead to more negative perceptions. Previous research indicating that men have higher perception of fertility and more favorable attitudes toward childbirth compared to women (24, 25) further supports this interpretation.

Although this study did not examine specific religious beliefs or practices, the findings align with previous research suggesting that religious affiliation is associated with a greater emphasis on childbearing, which may influence perceptions of infertility (26). In particular, religious and cultural groups that encourage childbearing often regard parenthood as a core element of personal identity. Members of such groups may experience distinct differences in how they perceive infertility as a personal challenge, their attitudes toward it, and their willingness to pursue infertility treatment (27). Qualitative work on religious women experiencing infertility has found that some interpret their childlessness in spiritual terms, for example as a test of faith or as God’s will, and describe feeling isolated from their religious community once others learn of their situation (28). A study of unmarried young adults in the US similarly found that those with a religious affiliation were more likely to believe they were personally infertile, even before marriage or any attempt to have children (29). Both findings point in the same direction: the pronatalist values often tied to religious affiliation may lead people to interpret infertility as personal failure and social stigma, consistent with how negative perception is defined in this study. Therefore, efforts to improve public perceptions of infertility should consider designing messages that harmonize with religious values and promote open dialog within religious communities to foster more inclusive and acceptable discourse.

This study also found that students who had been exposed to media addressing issues related to assisted reproductive technology (ART) exhibited more positive perceptions of infertility. This finding is consistent with prior studies showing that digital and social media-based sex and reproductive health education significantly enhances knowledge and attitudes (30–33). Media content that covers infertility treatments, success stories, and the need for support can help reduce stigma and encourage a more open and empathetic perspective on infertility. As patients increasingly turn to social media to seek, share, and utilize health-related information, understanding the infertility-related media landscape becomes increasingly important (34). Therefore, further research is needed to examine the specific types of media content and platforms that may shape perceptions of infertility.

For example, online infertility communities, YouTube vlogs, and Instagram hashtags serve as platforms for sharing personal experiences and offering emotional support. These spaces foster a sense of solidarity and understanding among individuals experiencing infertility, while also helping the general public cultivate empathy and understanding of infertility (35). As such, it is essential to normalize diverse family structures and reproductive health challenges through public media and education, and to frame infertility as a life event that anyone may experience. Practical approaches to this include public campaigns on reproductive health, integration of content into educational curricula, and the production of relevant broadcast and digital media content. In support of this, the high agreement among respondents regarding the acceptance of infertility as a natural part of life suggests a growing openness to discussing infertility and positioning it as a legitimate topic of public discourse.

In this study, factors influencing participants’ perceptions of infertility were analyzed, and gender stereotypes and family values were identified as significant predictors in the regression analysis. Specifically, the higher the degree of gender stereotypes and family values, the more negatively infertility tended to be perceived. A distinction is worth drawing here between these regression findings and the group differences identified through the t-test and ANOVA analyses (by gender, religious affiliation, and ART media exposure). The latter describe associations at the group level and should not be taken as evidence that these characteristics are modifiable. Gender stereotypes and family values, by contrast, are attitudinal constructs that could plausibly be addressed through education and counseling.

Gender stereotypes showed a negative correlation with infertility perception, suggesting that individuals with strong gender stereotypes are likely to have lower or distorted perceptions of infertility. In most cultures, women have traditionally been defined as mothers and wives, while men have been regarded as breadwinners (36). These gender stereotypes can function structurally to shift responsibility for infertility onto women, or to perceive men as unrelated to the issue. Recently, social pressure regarding marriage and childbirth has eased, and diverse lifestyles have gained acceptance, leading to a shift in how infertility is perceived—as an individual circumstance rather than a source of personal or social stigma. Although only a small proportion of young adults consider marriage and childbirth essential (37), gender roles remain divided according to patriarchal values, and gender inequality persists (25, 38). Previous studies on Korean university students also showed that although the value of marriage has changed over time, the roles of women and men remain divided according to patriarchal values, and gender inequality still exists (25, 38). Therefore, efforts to positively change infertility perception require approaches to resolve gender stereotypes and to strengthen gender-sensitive perspectives.

This study also found that the higher the family values, the more negative the perception of infertility, indicating that individuals who place high importance on the value of children and parenthood may perceive infertility as more fearful or negative. This aligns with previous research showing that in societies with strong pronatalist cultures, infertility is interpreted as social failure, which intensifies psychological distress and stigma (39, 40). In collectivist, family-centered cultures such as Korea, having children and fulfilling one’s role as a good son or daughter, and eventually a good parent, is closely tied to personal identity. As a result, losing the ability to have children can feel like a threat to one’s sense of self, not simply a medical issue (41). Therefore, it is necessary to provide emotion- and support-centered counseling considering family value levels, positive education on diverse family forms, and public campaigns including family members and spouses. This is expected to contribute to improving perceptions of infertility, alleviating fear, and promoting realistic and proactive coping strategies.

Interestingly, the subdomain analysis revealed a more nuanced relationship between family values and gender stereotypes than the overall correlation suggested. Specifically, family values were more strongly associated with familial and social gender roles than with intellectual or psychosocial gender roles. This pattern suggests that pronatalist values are more closely tied to gendered expectations regarding family and domestic roles than to broader psychological or intellectual gender stereotypes, which may have diluted the overall correlation. Subdomains were examined at the correlation stage to clarify the internal structure of each construct; however, only composite scores were entered into the regression model to avoid multicollinearity, consistent with prior use of these instruments.

Currently, the Korean government is implementing policies to address the low birth rate by providing financial support for artificial reproductive technology, antenatal care, and childcare (42). For these policies to be effective, people need to feel able to seek help without fear of stigma. Positive perceptions of infertility and a willingness to engage proactively with available support may encourage timely treatment-seeking and contribute to better reproductive health outcomes. This calls for public health efforts that go beyond service provision to include promotion of reproductive health literacy and broader awareness of treatment options, so that infertility is understood as a medical condition rather than a source of personal or social shame.

These findings suggest that educational and counseling programs aimed at promoting healthy reproductive attitudes among young adults need to do more than convey factual information; they should also address gender role awareness and openness to diverse family forms. Nevertheless, this study has several limitations. First, because the study was conducted among university students in a specific region, the generalizability of the results is limited. Second, the cross-sectional design restricts the ability to infer causal relationships among variables. Lastly, although the scale used to measure infertility perception (IPS-W) showed acceptable internal consistency for men, the lack of formal psychometric validation for the male minority group (*n* = 33) remains a methodological limitation.

## 5 Conclusion

This study demonstrates that university students’ perceptions of infertility are significantly influenced by gender stereotypes and family values, which can contribute to more negative perceptions. Given that university students are at a critical life stage with high reproductive potential, it is essential to support the development of more positive perceptions of infertility. Tailored educational programs that challenge rigid gender norms and respect diverse family values may help reduce stigma and fear associated with infertility. Further research using broader samples and longitudinal designs is needed to inform effective policies and interventions. Ultimately, fostering positive perceptions of infertility among young adults can promote healthier reproductive planning and support national efforts to address low birth rates.

## 6 Implications

This study highlights the importance of addressing gender stereotypes and family values when promoting infertility perception among young adults. For practice, university-level health education programs should incorporate gender-sensitive and media-supported content to foster more positive perceptions. For policy, public health campaigns should focus on university students and young adults to reduce stigma and enhance reproductive literacy. For research, future longitudinal and cross-cultural studies are needed to examine causal relationships and generalize findings to broader populations.

## Data Availability

The raw data supporting the conclusions of this article will be made available by the authors, without undue reservation.
